# Sequences of Mind Development in Boys with Autism Spectrum Disorder

**DOI:** 10.5402/2012/637453

**Published:** 2012-12-04

**Authors:** Abbas Bakhshipour, Majid Mahmood Aliloo, Hassan Shahrokhi, Toraj Hashemi, Shahrokh Amiri, Leila Mehdizadeh Fanid, Neda Yadegari, Farzin Hagnazari

**Affiliations:** ^1^Department of Psychology, Faculty of Psychology and Educational Sciences, University of Tabriz, 29 Bahman Bolvard, Tabriz 5166616471, Iran; ^2^Clinical Psychiatry Research Centre and Razi Hospital, Elgoli Road, Tabriz, East Azerbaijan 51664, Iran; ^3^Department of Psychiatry, Clinical Psychiatry Research Centre, Tabriz University of Medical Science, Razi Hospital, Elgoli Road, Tabriz, East Azerbaijan 51664, Iran; ^4^Department of Psychology, University of Tabriz and Autism Association, 29 Bahman Bolvard, Tabriz 5166616471, Iran

## Abstract

Autism is a pervasive neurodevelopment disorder, primarily encompassing difficulties in the social, language, and communicative domains. One of the most common social cognitive theories of autism is based on theory of mind (ToM), the “mentalizing” ability needed to infer that others have their own beliefs and desires in order to understand their behavior. In the current study, this hypothesis was tested using Wellman and Liu's scaled ToM tasks. These were employed in the assessment of ToM development of verbal, school-aged high-functioning boys with autism spectrum disorder (ASD). The results indicated that children with ASD performed significantly worse than normal children on ToM tasks (*Z* = 4.7; *P* < 0 .001). However, it was shown that some of the ASD children were able to pass desire and false-belief tasks whereas none of them could succeed in knowledge and real-apparent emotion tasks.

## 1. Introduction

Autistic spectrum disorders (ASDs) are neurodevelopmental disorders of unknown etiology with characteristic deficits in social interaction, communication, and behavior. One popular theory to explain the social skill deficits of subjects with autism is that they show severe impairments in the ability to attribute beliefs to themselves and others, that is, in their “theory of mind.” Theory of mind (ToM) refers to the ability to attribute internal mental states, such as beliefs, desires, and intentions, to oneself and others and to use those attributions to comprehend and predict behavior [1, 2]. 

The theory of mind hypothesis hypothesizes that autism involves impairment in the ability to conceive of mental states and to use mental state concepts to interpret and approximate one's own and other people's behavior [3]. Although attempts to stipulate the nature of the “metalizing” impairment in autism have increasingly taken a developmental rather than a static, all or nothing approach [4], the bulk of the research on theory of mind in autism has nonetheless focused on the attainment of one key social-cognitive milestone, false-belief understanding, in which individuals with autism have been found to be significantly impaired [5, 6].

 The ability to recognize false beliefs to oneself and others, which is normally acquired at around age 4, is considered a particularly important development in theory of mind in that it indicates the emergence of a representational concept of mind, whereby children implicitly understand that mental states are subjective representations of the world that are independent of and not necessarily congruent with reality [7–9]. From the advantage point of the theory of mind hypothesis, an impaired ability to represent mental states, and the limited awareness of oneself and other people that this implies, it gives a compelling elucidation for the failures in communication and reciprocal social interaction that characterize autism [10–12].

Various ToM tasks have been designed and applied to determine attribution of internal mental states. Complexity of these attributions has been categorized theoretically into first-order, second-order, third-order, and “advanced” ToM [13–15]. ToM tasks of varying levels of difficulty have been developed to explore these different levels of ToM ability in individuals with ASD. First-, second-, and third-order ToM tasks typically measure understanding of false beliefs, while other “advanced” ToM tasks measure a variety of other forms of mental state understanding. Researchers describe these tasks as “advanced” to indicate that they are likely more challenging for higher-functioning individuals with ASD than the first- and second-order ToM tasks commonly used in studies with this population [13].

However, it should be taken into account that about 20% to 25% of high-functioning individuals with autism pass false-belief tasks [16, 17]. These data generate several questions. For example, are individuals with autism distinctively impaired in theory of mind understandings or only significantly delayed? In response, Wellman and Liu [18] developed a set of scaled ToM tasks, which are designed to assess children's understanding of desires, emotions, knowledge, and beliefs. A scaled set of tasks may have several advantages. It could more comprehensively capture children's developing understandings across a range of conceptions. Establishing sequences of development would help constrict theorizing about theory of mind development. Moreover, a scaled set of tasks could provide a better measure to use in individual differences research examining the interplay between theory of mind understanding and other factors. This would include both the role of independent factors (e.g., family conversations, language, and executive functioning) on theory of mind and the role of theory of mind as an independent factor contributing to other developments (e.g., social interactions, peer acceptance) [19].

Hence, in the current research, the scaled ToM tasks [18] were adapted to Persian language and employed in the assessment of ToM development in high-functioning children with autism spectrum disorder. Subsequently, the main aim of this study was to assess high-functioning autism spectrum children's understanding of desires, knowledge, beliefs, and emotions.

## 2. Methods

To assess all components of ToM, 15 boys with high functional autism spectrum disorder, aged from 6 to 13, were selected from possible 85 autistic children. 

## 3. Participants

All subjects with ASD were selected from Tabriz Autism Association, which is the only organization available in east of Iran. They were then diagnosed with ASD following a detailed psychiatric assessment, developmental history, and a review of the data provided by their teachers and parents. These subjects were then examined and assessed by another psychiatrist, and at the end only 15 boys out of possible 85 subjects fulfilled the DSM-IV criteria for ASD [20]. Oral and written informed consents were obtained from at least one parent of all participants, and the research protocol was approved by the ethics committee of Tabriz University of Medical sciences. 

For control group, 15 volunteers were recruited from local school (15 boys) in the same age range. They were also examined to rule out any neurological, psychiatric, or learning problems. Furthermore, none of these children was on medication, and this information was gathered from one of their parents. 

## 4. Measures

All measures were administered by experienced clinical psychologist and cognitive neuroscientist in few visits scheduled. The full Wechsler Intelligence Scale for Children-Revised (WISC-R) was used to obtain IQ scores of all subjects. Only ASD children who had the total IQ score above 70 were chosen. The participants were group-wise matched on the basis of gender, chronological age, education, and full scale IQ. WISC-R was adapted and standardized for Iranian children by Shahim [21]. After full diagnostic assessments, IQ test was completed. Then separate batteries of theory of mind tasks were administered in counterbalanced order. Within each battery, individual tasks were administered in randomized order. Children's responses were scored during the assessment.

## 5. ToM Tasks

In this study, scaled ToM tasks [18] were employed to assess children's ToM ability. As it was mentioned previously, these tasks were categorized into four scales. The original ToM scale of Wellman and Liu was translated and adapted to Persian. Furthermore, to make these tasks understandable for Iranian children, minor changes were made (e.g., name of the characters in the story, etc.). Reliability (Cronbach's alpha) for the ToM tasks was 0.86. These standard tasks were then administered to assess children's understanding of desire, knowledge, emotion, and belief.  Table 1 summarizes scaling of theory of mind tasks, which are designed to measure various aspects of mind development in children. The full descriptions of all ToM tasks are listed in the appendices.

## 6. Statistical Procedures

To compare control measures (age, IQ) between ASD and control subjects, independent sample *t*-test was utilized, and to equate the level of education in both ASD and control group, chi-square test was applied. Furthermore, to evaluate the theory of mind ability of ASD and control subjects, chi-square test was also employed. To compare the total scores of ToM amongst both groups, Mann-Whitney *U* test was applied. Moreover, one-way ANOVA was computed to determine significant differences between ASD and control groups on verbal fluency and working memory measures. Finally, to study the relation between ToM ability and other components such as verbal fluency and working memory, Pearson's correlation was employed. 

## 7. Results

Demographic information for the samples is provided in Table 2. The sample included 15 boys with ASD. In addition, 15 control boys were selected to match children in ASD group for age, sex, IQ, and education. Two-tailed independent *t*-test results showed that there were not any significant differences between the ASD and control groups in terms of age (*t* = 0.3, df = 28, *P* > 0.05) and IQ (*t* = 0.121, df = 28, *P* > 0.05).

To compare the level of education in both ASD and control groups, chi-square test was applied (shown in Table 3). No significant variation in the level of education (*χ* ^2^ = 1.27, *P* > 0.05) between both groups was observed.

**Figure 1:**
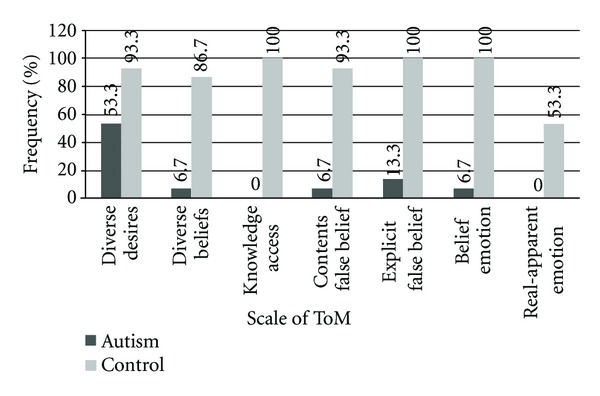
Evaluation of the results of scaled ToM tasks amongst ASD and control groups.

To evaluate the results obtained from scaled ToM tasks amongst ASD and control subjects, chi-square test was used. The results (as illustrated in Table 4 and Figure 1) were as follows.

- In the scale of desire, two types of test were employed, and these were diverse desire and diverse beliefs tasks. In diverse desire task, the percentage of negative answers in ASD subjects was 46.6%, and the percentage of right answers was 53.3%, whilst in control subjects, 93.3% right responses were given to the questions, and only 6.6% of the responses were wrong. According to chi-square test there was a significant difference (*χ* ^2^  =  6.14, *P* < 0.05) between the right and wrong responses amongst both groups. In diverse beliefs task, the percentage of negative answers in ASD subjects was 93.3%, and the percentage of right answers was 6.6%. In comparison, control subjects had 86.6% right responses and only 13.3% wrong answers. Chi-square test illustrated a significant difference (*χ* ^2^ = 19.29, *P* < 0.001) between the right and wrong responses amongst both groups.
- In the scale of knowledge, ASD subjects performed very poorly, and all gave wrong answers to the questions of this task while control subjects gave 100% right responses. According to chi-square test, there was a significant difference (*χ* ^2^ = 30, *P* < 0.001) between the responses amongst both groups.
- In the scale of belief, two tasks were applied, which were content false belief and explicit false belief. In content false-belief task, ASD children gave 93.3% wrong responses to the questions, and only 6.6% of the answers were true. Chi-square test indicated that there was a significant difference (*χ* ^2^ = 22.53, *P* < 0.001) between these groups. In explicit false-belief task, the percentage of right responses of ASD subjects was 13.3%, whereas control subjects scored 100%. According to chi-square test there was a significant difference (*χ* ^2^ = 21.99, *P* < 0.001) between the right and wrong responses amongst both groups.
- In the scale of emotion, two tests were also utilized, which were belief emotion and real-apparent emotion. In belief emotion task, the percentage of right answers in ASD group was 6.6%, while control subjects gave 100% right responses to the questions. According to chi-square test there was a significant difference (*χ* ^2^ = 26.25, *P* < 0.001) between the right and wrong responses amongst both groups. In real-apparent emotion task, ASD subjects did not give any right response to the questions and in control group, only 53.3% of the responses were true. However, according to chi-square test, there was a significant difference (*χ* ^2^ = 10.9, *P* < 0.001) between the right and wrong responses amongst both groups.

**Figure 2:**
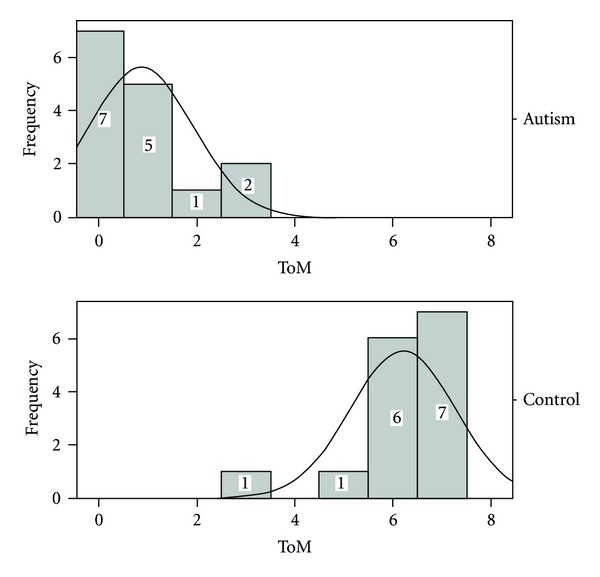
Histogram of total ToM scores of ASD and matched control groups.

According to scaled ToM tasks, the total scores that one can obtain from these tests are 7  (right responses). Therefore, the total scores obtained for each subject were also determined (see Table 5 and Figure 2). The mean total score for ASD subjects is 8.2, while ASD subjects have a mean total of 0.68. Nonparametric analyses (Mann-Whitney *U* test) showed that children with autism performed significantly worse than typically developing children on ToM tasks (*Z* = 4.7, *P* < 0.05). On the other hand, because the total scores obtained by ASD subjects were quite low (about 0.8), this shows that these children probably do not have the ToM ability.

## 8. Discussion

Children with autism exhibit deficient social interaction and communication skills, an unusual insistence on regularity, and abnormal adherence to repetitive patterns of behavior [20]. With respect to social understanding, specifically, there is now an agreement that children with autism show deficits on tasks that assess theory of mind [22]. 

ToM skills are fundamental to our understanding and assigning of mental states to self and others [1]. According to the well-known ToM account of autism [5], even the most high-functioning children with autism develop only low-level ToM skills. This is in stark contrast to typically developing children who acquire an elementary understanding of mental states by age two, with further development in the preschool years [23, 24]. Without an intact ToM, children with autism have difficulty using mental states to predict and explain others' behavior. As a result, they are developmentally delayed in their ability to communicate with others, to form relationships, and to make sense of their social environment [25].

This study addresses the sequence of understandings evident in ASD children's developing theory of mind. Wellman and Liu [18] have designed a set of tasks of increasing difficulty to measure ToM ability, but this has not been thoroughly used to assess autistic children or to compare autistic and normal children. The purpose of this study was to study the Wellman's ToM scale in more detail and to use it to compare ToM ability in two groups of ASD and normal children.

According to the results, some of the ASD children were able to pass the diverse desire and explicit false-belief tasks. For the first desire task, more than half of the ASD children managed to give the right responses, and only some of them were successful in explicit false-belief test. For some of the later tasks especially on knowledge access and real-apparent emotion, none of them could pass the tests. It is interesting to note that Peterson et al. [26], had similar findings even though, the ToM ability among autistic children in Peterson et al.'s [26] sample was higher than this study. However, their sample of autistic children was larger (*N* = 36) and older (mean and SD for age in months are 112 and 23, resp.). The result of this study showed that ASD children had more difficulty passing the test as they were getting more complex. Specially, on the scale of emotion, they could not really imagine another person's feeling in particular circumstances, simply because they could not put themselves in their shoes. However, the overall results suggest that ASD children's ToM ability is not totally absent and perhaps it can develop gradually and slowly. However, they had an overall poor performance on ToM tasks suggesting that they have a deficit in their theory of mind ability. The present study has succeeded in confirming and extending the conclusions drawn on the basis of the experiments by Baron-Cohen et al. [5] and Leslie and Frith [27]. It supports the claim that able autistic children are severely impaired in their theory of mind. Indeed, the present results underline just how poor autistic performance in understanding ToM tasks is. Certain aspects of theory of mind are more easily developed than others. This is true for the normal as well as the autistic child. One aspect concerns understanding informational access, for instance, understanding that one knows something because one has seen it and, conversely, that one does not know something because one has not seen it. 

However, this research can be continued in various ways. One possible area for further research is to conduct detailed item analysis (e.g., Guttman, Rasch, IRT, etc.) to investigate the developmental patterns in terms of item difficulties. In addition, more samples on autistic children with different stages of development are needed. This study of autistic children in Iran has provided additional and different samples from a different culture and educational system but perhaps repeats the claim that autistic children do have ToM abilities, which develop at a different pace and in different ways.

## Figures and Tables

**Table 1:** Scaling of theory of mind tasks [18].

Scale of ToM	Task	Description
Desire	Diverse desires	Child judges that two persons (the child versus someone else) have different desires about the same objects.
	Diverse beliefs	Child judges that two persons (the child versus someone else) have different beliefs about the same object, when the child does not know which belief is true or false.
Knowledge	Knowledge access	Child sees what is in a box and judges (yes-no) the knowledge of another person who does not see what is in a box.
Beliefs	Contents false belief	Child judges another person's false belief about what is in a distinctive container when child knows what it is in the container.
	Explicit false belief	Child judges how someone will search, given that person's mistaken belief.
Emotion	Belief emotion	Child judges how a person will feel, given a belief that is mistaken.
	Real-apparent emotion	Child judges how a person will feel, given a belief that is mistaken.

**Table 2:** Demographic data for the autism and control groups.

	*N*	Autism group		Control group		*t*	*P* value
		*M*	SD	*M*	SD		
Age	15	103.85	26.38	104.92	26.58	0.3	0.76
IQ	15	83.92	9.03	84.23	9.20	0.121	0.90

**Table 3:** Comparison of education level in ASD and control groups.

	Autism group		Control group		Chi-square tests	
	Count	%	Count	%	Value	*P* value
0	4	26.67	4	26.67		
1	3	20.00	3	20.00		
2	7	46.67	6	40.00	1.27	0.866
3			1	6.67		
5	1	6.67	1	6.67		

Total	15	100	15	100		

0: preschool, 1: first grade, 2: second grade, 3: third grade, and 5: fifth grade (elementary school grades in Islamic Republic of Iran).

**Table 4:** Evaluation of the Results of scaled ToM tasks amongst ASD and control groups.

		Group	Negative		Positive		Chi-square tests	
			Count	%	Count	%	Value	*P* value
Desire	Diverse desires	Autism	7	46.67	8	53.33	6.14	0.01*
		Control	1	6.67	14	93.33		
	Diverse beliefs	Autism	14	93.33	1	6.67	19.29	0.00**
		Control	2	13.33	13	86.67		
Knowledge	Knowledge access	Autism	15	100			30	0.00**
		Control			15	100		
Beliefs	Contents false belief	Autism	14	93.33	1	6.67	22.53	0.00**
		Control	1	6.67	14	93.33		
	Explicit false belief	Autism	13	86.67	2	13.33	21.99	0.00**
		Control			14	100		
Emotion	Belief emotion	Autism	14	93.33	1	6.67	26.25	0.00**
		Control			15	100		
	Real-apparent emotion	Autism	15	100			10.9	0.001**
		Control	7	46.67	8	53.33		

^*∗∗*^Significant at the 0.01 level ( *P* < 0.01).

^*∗*^Significant at the 0.05 level ( *P* < 0.05).

**Table 5:** Assessment of total ToM scores of ASD and matched control subjects.

Group	*N*	*M*	SD	Min	Max	Mann-Whitney *U* test	
						*Z*	*P* value
Autism	15	0.86	1.06	0	3	4.71	0.000*
Control	15	6.2	1.08	3	7		

*Significant at the 0.05 level (*P* < 0.05).

## Appendices

### A. Diverse Desires

Children see a picture of a boy called Amir and then a sheet of paper with a carrot and a cookie drawn on it. “Here's Amir. It's snack time, so, Amir wants a snack to eat. Here are two different snacks: a carrot and a cookie. Which snack would you like best? Would you like a carrot or a cookie best?” This is the own desire question. If the child chooses the carrot: “Well, that's a good choice, but Amir really likes cookies. He does not like carrots. What he likes best are cookies.” (Or, if the child chooses the cookie, he or she is told Amir likes carrots.) Then the child is asked the target question: “So, now it's time to eat. Amir can only choose one snack, just one. Which snack will Amir choose? A carrot or a cookie?” To be scored as correct or to pass this task, the child must answer the target question opposite from his or her answer to the own desire question. This task was derived from those used by Wellman and Woolley [28] and Repacholi and Gopnik [29].

### B. Diverse Beliefs

Children see a toy figure of a girl and a sheet of paper with bushes and a garage drawn on it. “Here's Sara. Sara wants to find her ball. Her ball might be behind the bushes or it might be in the garage. Where do you think the ball is? Behind the bushes or in the garage?” This is the own belief question. If the child chooses the bushes: “Well, that's a good idea, but Sara thinks the ball is in the garage. She thinks her ball is in the garage.” (Or, if the child chooses the garage, he or she is told Sara thinks her ball is behind the bushes.) Then the child is asked the target question: “So where will Sara look for her ball? Behind the bushes or in the garage?” To be correct the child must answer the target question opposite from his or her answer to the own belief question. This task was derived from those used by Wellman and Bartsch [30] and Wellman et al. [31].

### C. Knowledge Access

Children see a plain plastic box with a drawer containing a small plastic toy dog inside the closed drawer. “Here's a drawer. What do you think is inside the drawer?” (The child can give any answer he or she likes or indicate that he or she does not know.) Next, the drawer is opened, and the child is shown the content of the drawer: “Let's see, it's really a dog inside!” Close the drawer: “Okay, what is in the drawer?” Then a toy figure of a girl is produced: “Zahra” has never ever seen inside this drawer. Now here comes Zahra. So, does Zahra know what is in the drawer? (The target question.) “Did Zahra see inside this drawer?” (The memory question.) To be correct the child must answer the target question “no” and answer the memory control question “no.” This task was derived from those used by Pratt and Bryant [32] and Pillow [33], although it was modified so that the format was more parallel to the contents false-belief task.

### D. Contents False Belief

The child sees a clearly identifiable pencil case with a plastic toy rabbit inside the closed pencil case. “Here's a pencil case. What do you think is inside the pencil case?” Next, the pencil case is opened: “Let's see, it's really a rabbit inside!” The pencil case is closed: “Okay, what is in the pencil case?” Then a toy figure of a boy is produced: “Ali” has never ever seen inside this pencil case. Now here comes Ali. So, what does Ali think is in the box? Pens and pencils or a rabbit? (The target question.) “Did Ali see inside this box?” (The memory question.) To be correct the child must answer the target question “pens or pencils” and answer the memory question “no.” This task was derived from one used initially by Perner et al. [34] and widely modified and used since then.

### E. Explicit False Belief

Children see a toy figure of a boy and a sheet of paper with a backpack and a closet drawn on it. “Here's Reza. Reza wants to find his mittens. His mittens might be in his backpack or they might be in the closet. Really, Reza's mittens are in his backpack. But Reza thinks his mittens are in the closet.” “So, where will Reza look for his mittens? In his backpack or in the closet?” (The target question.) “Where are Reza's mittens really? In his backpack or in the closet?” (The reality question.) To be correct the child must answer the target question “closet” and answer the reality question “backpack.” This task was derived from one used by Wellman and Bartsch [30] and Siegal and Beattie [35].

### F. Belief** **Emotion

Children see a toy figure of a boy and a clearly identifiable individual-size Smartis box with rocks inside the closed box. “Here is a Smartis box and here is Teddy. What do you think is inside the Smartis box?” (Smartis.) Then the adult makes Teddy speak: “Teddy says, ‘Oh good, because I love Smartis. Smartis are my favorite snack. Now I will go play.” Teddy is then put away and out of sight. Next, the Smartis box is opened, and the contents are shown to the child: “Let's see that there are really rocks inside and no Smartis! There's nothing but rocks.” The Smartis box is closed: “Okay, what is Teddy's favorite snack?” (Smartis.)

Then Teddy comes back: “Teddy has never ever seen inside this box. Now here comes Teddy. Teddy's back and it's snack time. Let's give Teddy this box. So, how does Teddy feel when he gets this box? Happy or sad?” (The target question.) The adult opens the Smartis box and lets the toy figure look inside: “How does Teddy feel after he looks inside the box? Happy or sad?” (The emotion-control question.) To be correct, the child must answer the target question ‘‘happy” and answer the emotion-control question “sad.” This task was derived from one used by Harris et al. [36].

### G. Real-Apparent Emotion

Initially, children see a sheet of paper with three faces drawn on it a happy, a neutral, and a sad face, to check that the child knows these emotional expressions. Then that paper is put aside, and the task begins with the child being shown a cardboard cutout figure of a boy drawn from the back so that the boy's facial expression cannot be seen. “This story is about a boy. I'm going to ask you about how the boy really feels inside and how he looks on his face. He might really feel one way inside but look a different way on his face. Or, he might really feel the same way inside as he looks on his face. I want you to tell me how he really feels inside and how he looks on his face.” “This story is about Hamid. Hamid's friends were playing together and telling jokes. One of the older children, Hassan, told a mean joke about Hamid and everyone laughed. Everyone thought it was very funny, but not Hamid. But, Hamid did not want the other children to see how he felt about the joke, because they would call him a baby. So, Hamid tried to hide how he felt.”

Then the child gets two memory checks: “What did the other children do when Hassan told a mean joke about Hamid?” (Laughed or thought it was funny.) “In the story, what would the other children do if they knew how Hamid felt?” (Call Hamid a baby or tease him.) Pointing to the three emotion pictures: “So, how did Hamid really feel, when everyone laughed? Did he feel happy, sad, or okay?” (The target-feel question.) “How did Hamid try to look on his face, when everyone laughed? Did he look happy, sad, or okay? (The target-look question.) To be correct the child's answer to the target-feel question must be more negative than his or her answer to the target-look question (i.e., sad for target-feel and happy or okay for target-look, or okay for target-feel and happy for target-look). This task was derived from one used by Harris et al. [37].
